# Conversion of Non-Optical Material to Photo-Active Nanocomposites through Non-Conventional Techniques for Water Purification by Solar Energy

**DOI:** 10.3390/molecules25194484

**Published:** 2020-09-30

**Authors:** Osama Saber, Adil Alshoaibi, Mohammed Al-Yaari, Mostafa Osama

**Affiliations:** 1 Department of Physics, College of Science, King Faisal University, P.O. Box 400, Al-Ahsa 31982, Saudi Arabia; aalshoaibi@kfu.edu.sa; 2 Egyptian Petroleum Research Institute, Nasr City, P.O. Box 11727, Cairo 11765, Egypt; 3 Chemical Engineering Department, College of Engineering, King Faisal University, P.O. Box 380, Al-Ahsa 31982, Saudi Arabia; malyaari@kfu.edu.sa (M.A.-Y.); mostafa.osama8664@gmail.com (M.O.)

**Keywords:** alumina-nanocomposites, explosive processes, fast removal of green pollutants, low band gap energy, solar energy

## Abstract

Development of optical materials has attracted strong attention from scientists across the world to obtain low band gap energy and become active in field of solar energy. This challenge, which cannot be accomplished by the usual techniques, has overcome through the current study using non-conventional techniques. This study has used explosive reactions to convert non-optical alumina to series of new optical nanocomposites with very low band gap energy for the first time. In this trend, alumina nanoparticles were prepared and modified by explosive reactions using ammonium nitrate as a solid fuel. By using methanol or ethanol as a source of carbon species, three nanocomposites were produced indicating a gradual reduction of the band gap energy of alumina from 4.34 eV to 1.60 eV. These nanocomposites were obtained by modifying alumina via two different carbon species; core-shell structure and carbon nanotubes. This modification led to sharp reduction for the band gap energy to become very sensitive in sunlight. Therefore, these nanocomposites caused fast decolorization and mineralization of green dyes after illuminating in sunlight for ten minutes. Finally, it can be concluded that reduction of the band gap energy introduces new optical materials for developing optical nano-devices and solar cells.

## 1. Introduction

Alumina considers one of the most familiar advanced materials in the field of structural engineering materials [1]. Its low dielectric loss, coupled with its electrically insulating nature, reasonably low dielectric permittivity, and moderate thermal conductivity offered many applications that comprise electronic substrates for integrated circuits [1]. Bulk alumina is different from its nanoparticles because it has wide band gap energy in the range of 8.7–9.4 eV. Therefore, it can be used as an insulator. While, the band gap energy of the alumina nanoparticles decreased to lower values because of the existence of defect states located in the band gap region [2,3,4,5,6].

To produce highly optical properties with unique surface texture, many attempts have used to develop the structures of semiconductor materials. Nano alumina could be a good candidate for this purpose because the alumina nanoparticles showed some optical properties not found in its bulk phase. Shamala et al. [7] indicated that the band gap energy decreased to 5.75 eV in case of the evaporated film of the alumina nanoparticles. While, for spray deposited film, the optical band gap decreased to be 5.40 eV. Amirsalari and Shayesteh [1] reported that conversion of alumina from the bulk state to the nanoparticles caused reduction for the band gap energy from 9.0 eV to 4.50 eV because of the defect levels located in the optical band gap energy. Therefore, they recommended that alumina in the nano scale may be useful for many applications.

Water pollution and deficient energy have emerged as major challenges for scientific community because of the rapidly growing industries and population. Scientists across the world have focused on discovery methods to solve these energy and environment related problems. A considerable amount of research work has been carried out on producing non-polluting energy for purifying water. Sunlight is a non-polluting source for energy. The solar energy can be used to purify water by converting the industrial pollutants to carbon dioxide and water through photocatalytic degradation of green dyes. Following this trend, the current study has used carbon species to reduce the band gap energy of alumina to be effective in sunlight for purifying water from colored pollutants.

An incorporation of carbon nanotubes (CNTs) might be useful for narrowing the band gap energy of alumina. CNTs have a large surface area consisting of rolled up sheets of graphite with few microns in length and several nanometers in thickness. Carbon nanotubes are composed of sp^2^ bonded carbon atoms. These nanotubes biologically and chemically are more effective than graphite because the curving of the CNTs leads to the orbitals to slightly delocalize outside the tubes [8]. The reactivity of CNTs makes them a good choice for enhancing the optical properties of porous alumina.

There are two critical problems for the reinforcement process of alumina using carbon nanotube through the conventional mixing techniques between CNTs and alumina species [9]. However the conventional mixing is the only available method for producing CNTs based nanocomposites because of the high temperature of growth and production of CNTs through CVD or plasma techniques. The agglomeration behavior of CNTs, due to the van der Waals attractions between their sidewalls, is the first problem. Growth of the interface connection between the alumina matrix and CNTs that produced better properties for the nanocomposite considers the second problem [8]. These problems were avoided by many authors through creating functional groups for the sidewalls of CNTs [8,9]. To functionalize CNTs, concentrated sulfuric acid and nitric acid were commonly used for changing the interaction characteristics of the nanotubes and making them hydrophilic through removing non-wetting properties of CNT surfaces to be easily dispersed in the medium of reaction. However, this method caused other problems for the produced nanocomposites through creating a high quantity of defects because of damage and loss in the surface of CNTs [8].

The best way for obtaining a good homogeneous dispersion of carbon nanotubes in the matrix of alumina is a growth of CNTs during construction of hydrated alumina matrix. It means that CNTs must be produced at a low temperature similar to production of alumina matrix. It can be achieved through non-conventional techniques such as explosive reactions.

In the present study, alumina species have prepared and saturated by explosive materials such as ammonium nitrate. This combination between aluminium species and ammonium nitrate considers a strong solid fuel. By this way, explosive technique has used for growing different carbon species through using different sources of carbon. This novel technique favors the growth of CNTs at lower temperature than the other methods. By controlling the explosive technique, alumina nanoparticles start to grow with different structures of carbon. To maximize the effect of the carbon source, three nanocomposites were prepared depending on changing the ratio between source of carbon and solid fuel. Additionally, alumina nanoparticles were prepared by the same technique without carbon source for comparison. The prepared materials are characterized by many tools. UV-Vis spectroscopy has been used for studying, in detail, the optical properties of the prepared nanomaterials. These optical properties were tested through using solar energy to purify the water from green dyes.

## 2. Results

### 2.1. Characterization of the Prepared Solid Fuel

**Figure 1:**
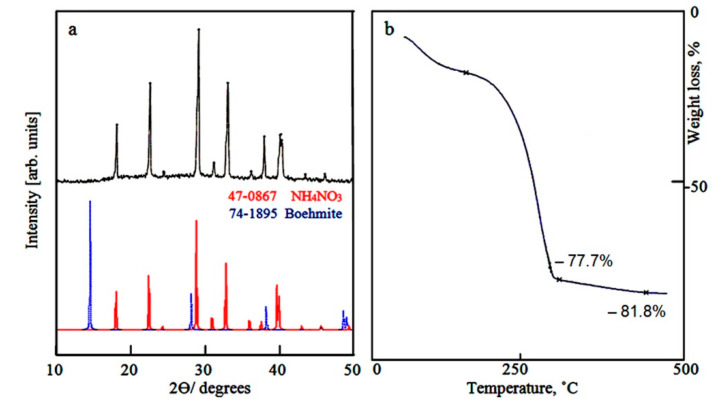
The dried product: (**a**) X-ray diffraction and (**b**) Thermal gravimetric analysis.

Figure 1a showed the X-ray diffraction pattern of the dried gel. Strong reflections were observed at 2θ = 17.98, 22.48, 28.94, 32.96, 36.14, 37.78, 39.86, 40.16, indicating that this structure contains ammonium nitrate in crystalline structure as reported in the JCPDS file No. 47–867. This finding can be explained according to the next reaction:

Al (NO_3_)_3_ + 3NH_4_HCO_3_ → 3NH_4_NO_3_ + Al (OH)_3_ + 3CO_2_ (1)

It means that the dried gel consists of ammonium nitrate and aluminium hydroxide. By comparing with the standard diagrams of boehmite (JCPDS file No. 74–195) and ammonium nitrate (JCPDS file No. 47–867), the peaks of aluminium hydroxide were not observed.

Thermal gravimetric technique is a suitable method for determining percentage of ammonium nitrate in the structure of the prepared gel because ammonium nitrate is completely decomposed with high temperature as shown in the following reaction:

8NH_4_NO_3_ → 5N_2_ + 4NO + 2NO_2_ + 16H_2_O (2)

The thermal gravimetric analysis (TGA) of the dried gel was shown in Figure 1b. The TG curve showed that weight loss of about 77.7 wt.% was observed at 300 °C. It means that the ratio between aluminium nitrate and aluminum hydroxide is 3:1 agreeing with the Equation (1).

### 2.2. Characterization of the Prepared Nanocomposites

**Figure 2:**
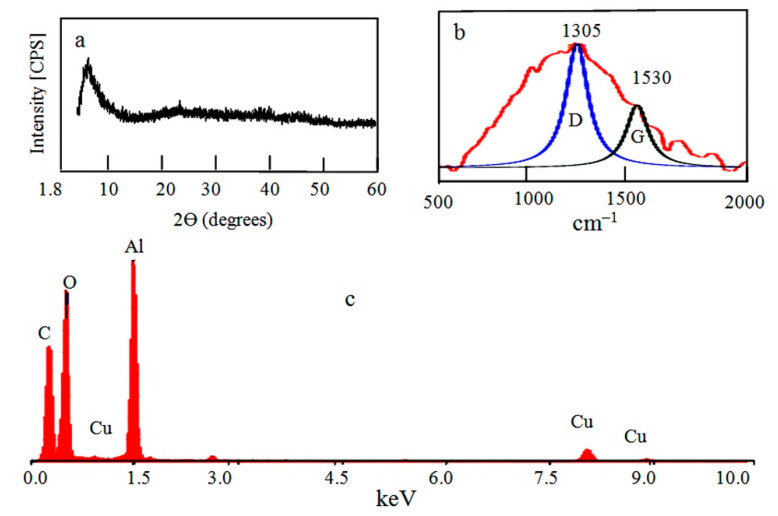
The prepared nanocomposite NC-1: (**a**) X-ray diffraction, (**b**) Raman spectrum, and (**c**) energy-dispersive X-ray spectrometry (EDX) analysis.

Three nanocomposites were obtained by reaction of the prepared solid fuel of aluminium hydroxide with alcohol as a source of carbon species. X-ray diffraction pattern of the first nanocomposite NC-1, which was produced using methanol as a source of carbon species, is displayed in Figure 2a. It showed one strong and broad peak at 2θ between 1.8° and 10°, indicating mesoporous amorphous alumina [10,11]. This peak was recorded at 1.75 nm (2θ = 5°). There are no other peaks recorded in the diagram indicating non-crystalline porous structure. Energy-dispersive X-ray spectrometry (EDX) analysis identified the different elements in the outermost parts of the nanocomposite NC-1. Aluminium, carbon, and oxygen were clearly recorded in the spectrum as shown in Figure 2c. It is known that the signal of the copper belongs to the copper substrate of the sample. The intensity peak of carbon was large indicating the high content of carbon species in the nanocomposite. It means that methanol is working as a source for producing carbon species.

Raman scattering has been widely used to characterize carbon nanotubes (CNTs) [12,13]. Raman spectrum exhibited one broad band in the range of 500–2000 cm^−1^ as shown in Figure 2b. CNTs have two bands in this range as reported in the results of Boscarino et al. [14]. These two bands are labeled as G and D band. They concluded that D and G bands of the ZnO-decorated CNTs were found at 1337 cm^−1^ and 1600 cm^−1^, respectively. Additionally, our previous study indicated that the LDH-CNTs have two bands at 1320 cm^−1^ (D band) and 1581 cm^−1^ (G band) [15]. In the current study, these “D” and “G” bands were observed at 1305 cm^−1^ and 1530 cm^−1^ by using Gaussian functions. The “D” band indicates disorder induced in the graphitic lattice or defects in carbon nanotubes. The “G” band is due to the tangential stretching modes of carbon nanotubes. The D and G bands were overlapped in a broad band and concentrated at 1305 cm^−1^ as shown in Figure 2b. This overlap indicates that NC-1 has low order of graphitic structure of CNTs. The broadness of the band could be explained by the results of Inbaraj et al. [16]. The experimental results of Inbaraj et al. indicated that alumina has bands in the Raman spectrum at 1373 cm^−1^ and 1403 cm^−1^. It means that the alumina bands are adjacent to the bands of CNTs in this small region of the spectrum. Therefore, the four bands were almost merged displaying a broad band. Additionally, this broad band indicates that NC-1 has well dispersed CNTs in the structure of alumina.

**Figure 3:**
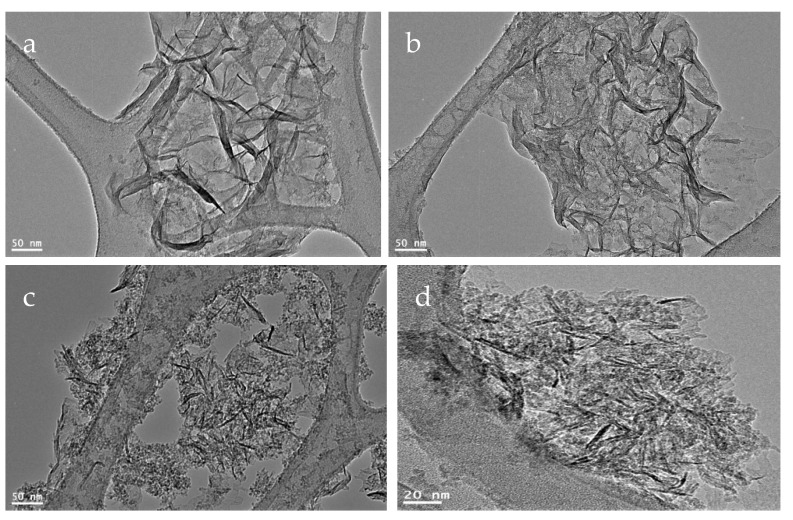
TEM images of the nanocomposite NC-1: (**a**,**b**) before thermal treatment and (**c**,**d**) after thermal treatment.

TEM images confirmed the well dispersion of CNTs inside the alumina texture as shown in Figure 3a,b. By calcination at 600 °C, Figure 3c showed that carbon nano-needles are dispersed inside alumina nanoparticles. While carbon nanotubes were observed in Figure 3d. It means that the prepared nanocomposite NC-1 is composed of carbon nanotubes and nanorods dispersed inside porous structure of alumina.

**Figure 4:**
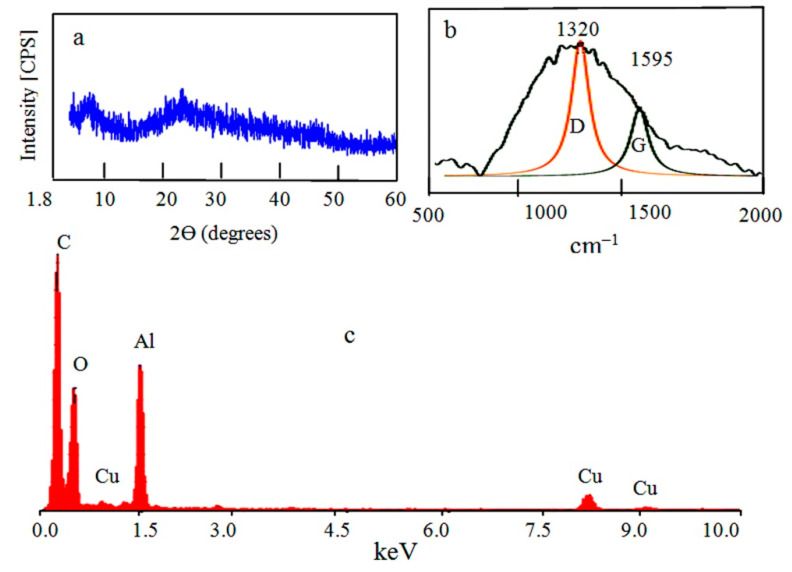
The prepared nanocomposite NC-2: (**a**) X-ray diffraction, (**b**) Raman spectrum, and (**c**) EDX analysis.

The second nanocomposite NC-2 was produced by using ethanol as a source of carbon species. Non-crystalline structure of NC-2 was observed as shown in Figure 4a. At low 2θ, weak peak was observed at the interval 1.8° and 10° indicating mesoporous amorphous alumina similar to the first nanocomposite. However, the weak peak represents lower porosity at 1.4 nm (2θ = 6.3°). Additionally, there is a weak peak observed at near 2θ = 22.58°. It was indexed as (002) plane indicating hexagonal graphite lattice in association with an existence of large quantity of amorphous carbon.

**Figure 5:**
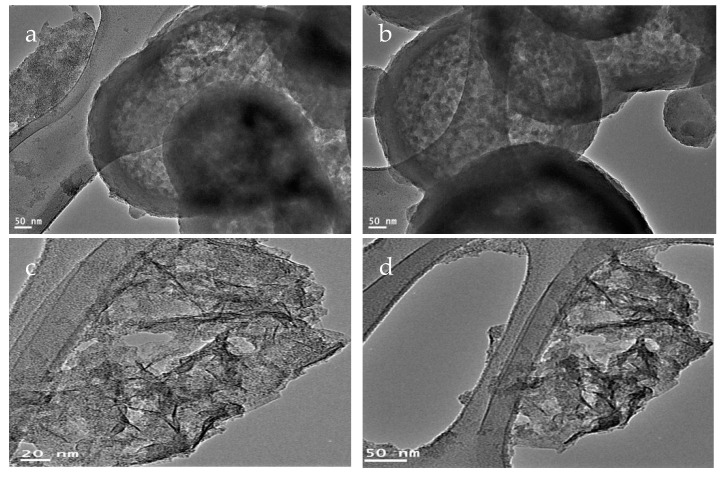
TEM images of the prepared nanocomposite NC-2 (**a**) first location, (**b**) second location, (**c**) third location and (**d**) fourth location.

By energy-dispersive X-ray spectrometry (EDX) analysis, the different elements in the second nanocomposite NC-2 were identified as shown in Figure 4c. Figure 4c revealed the high content of carbon species as strong peak in the spectrum. Aluminium oxide was also confirmed by the clear peaks of aluminium and oxygen in Figure 4c. It means that ethanol can act as a source of carbon species in this technique. Raman spectrum of NC-2 confirmed the presence of carbon species as CNTs. Where, Figure 4b showed the D and G bands that characterized for graphitic carbon nanomaterials. The main band was observed at 1320 cm^−1^ as D-band. It is due to disorder structure of carbon. The G band which indicates the graphitic sheets formation of CNTs was detected at 1595 cm^−1^. TEM images of NC-2 showed that the second nanocomposite NC-2 has core-shell structure as shown in Figure 5a,b. In the other locations, carbon nanotubes were observed in Figure 5c,d.

**Figure 6:**
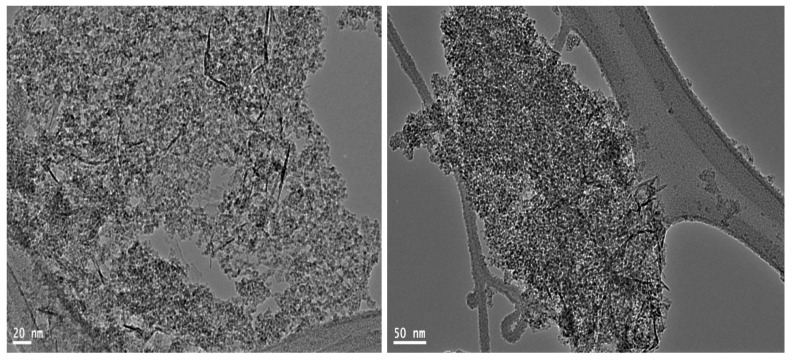
TEM images of the prepared nanocomposite NC-2 after thermal treatment.

These carbon nanotubes became clearer by calcination at 600 °C as shown in Figure 6. Additionally, Figure 6 showed network of aluminium oxide nanoparticles. These analyses concluded that the second nanocomposite has two structures. The first structure is aluminium oxide nanoparticles coated by nanolayers of amorphous carbon looking like core-shell structure. The second structure indicated that CNTs are homogeneously distributed through the matrix of aluminium oxide nanoparticles.

**Figure 7:**
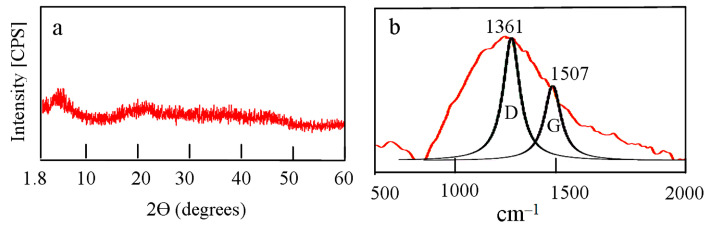
The prepared nanocomposite NC-3: (**a**) X-ray diffraction and (**b**) Raman spectrum.

By increasing the ratio of carbon source to solid fuel, the third nanocomposite NC-3 was produced through reducing the amount of solid fuel. X-ray diffraction of NC-3 is displayed in Figure 7a. It is similar to that of the second nanocomposite indicating amorphous structure. Raman spectrum of NC-3 showed the characteristic peaks of graphitic materials. Figure 7b revealed the D and G bands at 1361 cm^−1^ and 1507 cm^−1^, respectively. The broad band at the interval 700 cm^−1^–2000 cm^−1^ confirmed presence of amorphous carbon in addition to aluminium oxide nanoparticles. The similarity of X-ray patterns and Raman spectra between both the second and the third nanocomposites indicated that both of them have the same structure.

TEM images and Raman spectra indicated that the carbon source in the explosive reactions played an important role for building the structure of nanocomposites. In case of the first nanocomposite, NC-1, the using of methanol as a carbon source led to producing carbon nanotubes homogeneously dispersed in the matrix of aluminium oxides. While, by using ethanol as a source of carbon, two kinds of carbon species were produced and combined with aluminium oxides through two structures for building both nanocomposites NC-2 and NC-3. The first structure is composed of CNTs dispersed in the matrix of aluminium oxides and the second structure comprised of aluminium oxides nanoparticles coated with a thin film of carbon looking like core-shell structure.

### 2.3. Optical Properties

It is known that the bulk phase of alumina has a wide band gap energy in the range of 8.7–9.4 eV and is considered as an insulator. There have been many attempts to modify alumina structures to improve their optical properties. To investigate the optical behavior of the alumina nanoparticles and their nanocomposites, the UV-Vis absorption spectra have used as a powerful tool for providing important details about their absorbance and band gaps.

**Figure 8:**
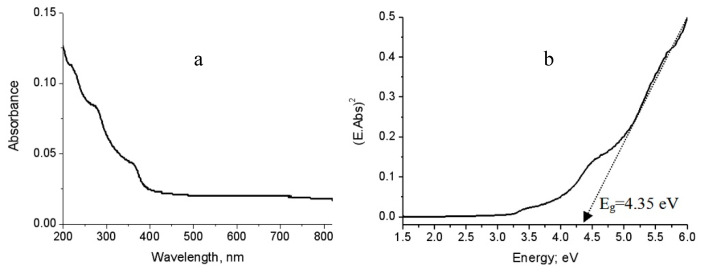
The prepared alumina nanoparticles: (**a**) Absorbance spectrum and (**b**) Band gap energy.

Figure 8a is displayed the UV-Vis absorbance of the pure aluminium oxide nanoparticles that was prepared without carbon species. It showed absorption edge near to 400 nm with three maxima at 220 nm, 290 nm and 360 nm. By comparing with the bulk aluminium oxide in which no absorptions are recorded within the spectral region 200–400 nm, the nano size plays positive role for improving the absorbance of alumina. These peaks were attributed to the intrinsic defect centers that have absorption bands in these regions [17].

By using absorbance data, the energy band gap (E_g_) could be calculated through the scientific relation between the absorbance coefficient of the materials (Abs) and the incident photon energy (E) according to the following equation [18]:

(E.Abs)^2^ = constant (E − E_g_) (3)

Where E is calculated by multiplying the Planck’s constant (h) with speed of light (c) and divided by the wavelength (λ). The absorption coefficient (Abs) describes the optical absorption process. The value (2) is used for the allowed direct transitions.

Therefore, the band gap energy of the prepared alumina could be determined by plotting both (E.Abs)^2^ and (E). By extending the straight line to the (E) axis, the optical band gap energy can be obtained when the value (E.Abs)^2^ equal zero as shown in Figure 8b. Figure 8b showed that the band gap energy of the prepared aluminium oxide nanoparticles is 4.34 eV. Comparing with the bulk aluminium oxide, the band gap energy of alumina decreased from 8.7 eV to 4.34 eV because of the positive effect of the nano size for producing defect states in the band gap region agreeing with the results of Amirsalari and Shayesteh [1].

**Figure 9:**
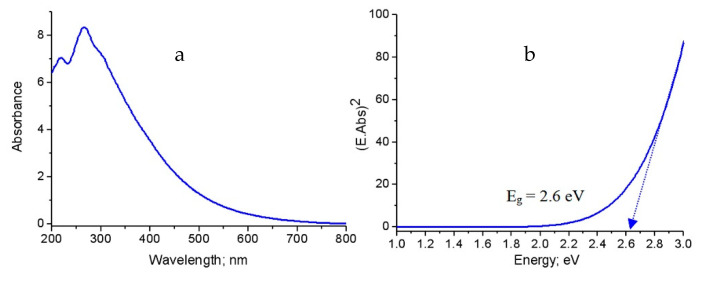
The prepared nanocomposite NC-1: (**a**) Absorbance spectrum and (**b**) Band gap energy.

**Figure 10:**
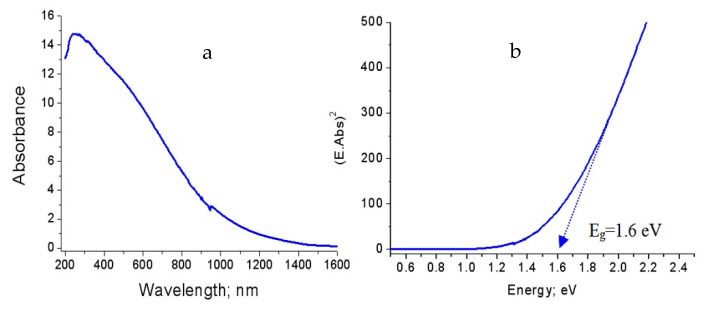
The prepared nanocomposite NC-2: (**a**) Absorbance spectrum and (**b**) Band gap energy.

**Figure 11:**
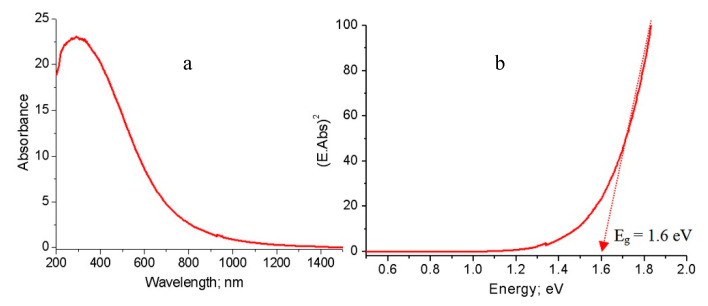
The prepared nanocomposite NC-3: (**a**) Absorbance spectrum and (**b**) Band gap energy.

By growing both carbon species and alumina nanoparticles through the explosive reactions of aluminium hydroxide, the optical properties of the prepared nanocomposites based on alumina are strongly modified as shown in Figure 9, Figure 10 and Figure 11.

Figure 9a displayed the absorbance of the first nanocomposite NC-1. It showed that the absorption edge shifted to 700 nm with the maximum intensity at 280 nm. Additionally, the absorbance spectrum became wider as shown in Figure 9a. It means that the material became active in the visible region. This finding was confirmed by determining the band gap energy. The band gap energy of NC-1 was 2.62 eV as seen in Figure 9b. This band gap energy was quite different from those of aluminium oxides bulk (E_g_ = 8.7 eV) [1] and nanoparticles (E_g_ = 4.35 eV). This large narrowing of the band gap energy may be explained according to the presence of new levels in the band gap region because of defect states of CNTs. This shrinkage is due to a consequence of many body effects on the conduction and the valence bands of aluminium oxides such as exchange energy due to electron–electron and electron-impurity interactions of CNTs. These effects lead to a narrowing of the band gap (red shift) [17].

In case of the second nanocomposite NC-2, Figure 10a reveals the absorbance spectrum in the wavelength range of 200–1500 nm. This broad absorbance indicates that the material became very sensitive for visible light. The calculated band gap energy of NC-2 confirmed this observation as shown in Figure 10b. It revealed 1.6 eV for the second nanocomposite. Compared to the band gap energy of both the first nanocomposite and alumina nanoparticles, more narrowing of band gap of alumina happened after changing methanol to ethanol as a source of carbon.

These observations were also noticed in the third nanocomposite NC-3 as shown in Figure 11. Figure 11a showed a broad absorbance of NC-3 in the wavelength range 200–1100 nm. It means that the third nanocomposite became optically active in the visible region. The band gap energy of NC-3 was 1.6 eV as shown in Figure 11b.

**Figure 12:**
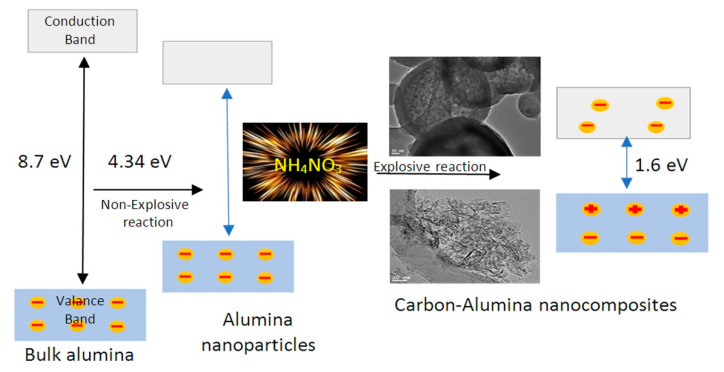
Schematic representation for converting bulk alumina to photo-active alumina.

Comparing with the band gap energy of the second nanocomposite, the third nanocomposite has similar effect in the visible region. This large narrowing of the band gap energy of the prepared nanocomposites is probably due to chemical interaction between CNTs and aluminium oxide nanoparticles, generating a new energy level to reduce the band gap energy [19,20]. The narrowing of the band gap energy of the second and third nanocomposites may be due to the growth of another type of carbon species in which the core-shell structure formed. The link between the core (aluminium oxide) and the shell (carbon nanolayers) generates other energy levels, decreasing the band gap energy. These experimental results are summarized in Figure 12 to indicate the conversion of bulk alumina from non-optical behavior to photo-active products.

### 2.4. Fast Removal of Pollutants

In order to test the solar activity of the prepared nanocomposites, the photocatalytic removal of the green dyes (naphthol green B; NGB) from water were studied using NC-1, NC-2, and NC-3 in the presence of sunlight. At the same time, to evaluate the effect of explosion reactions on the optical properties of alumina, the solar activity of the prepared nanoparticles of alumina was measured by the same process and compared with the solar activity of the nanocomposites.

The concentration of the green pollutants was determined by the intensity of the absorption of naphthol green B at λ_max_= 720 nm. A blank experiment indicated that NGB has high stability in sunlight because no variation in the concentration was noticed in sunlight.

**Figure 13:**
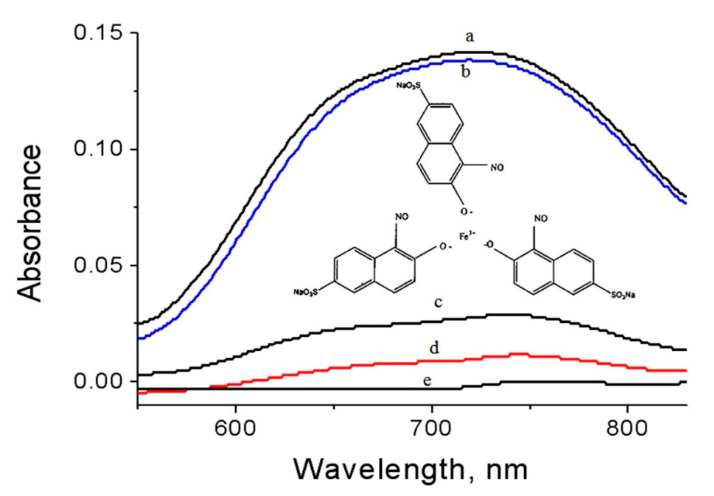
Absorbance spectra of naphthol green B after10 min in sunlight (**a**) No sample, and in presence of (**b**) alumina nanoparticles, (**c**) NC-1, (**d**) NC-2, and (**e**) NC-3.

**Figure 14:**
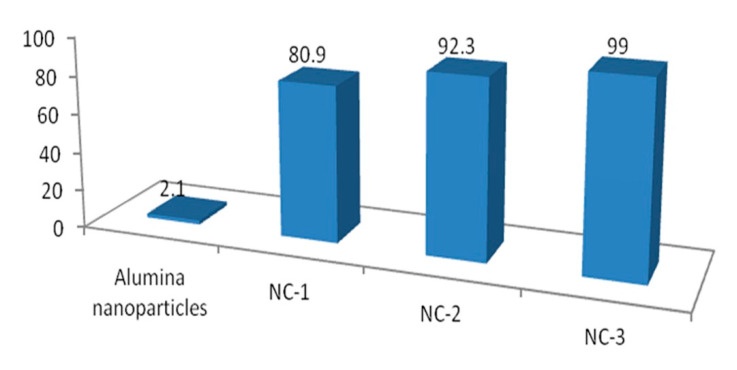
Removal percentage of naphthol green B after 10 min in sunlight.

A green mixture with one of the prepared materials was illuminated for ten minutes in sunlight. After that the concentration of the green dyes was determined through measuring the absorbance of the green solution. These results are displayed in Figure 13 and Figure 14. The percentage of naphthol green B in presence of NC-1 sharply decreased after illuminating 10 min in sunlight as shown in Figure 13. Figure 14 showed 80.9% removal of the green pollutant. In case of using NC-2, the removal increased to be 92.3% in the same period, while complete decolorization and removal of the green dyes were nearly achieved after using NC-3. Figure 13 showed approximately complete disappearance for the absorbance of the green dyes. Additionally, Figure 14 revealed that 99% of the green dyes was removed by NC-3 after irradiating 10 min in sunlight. In case of using the nanoparticles of alumina in sunlight, slight variation was noticed for the intensity of the absorbance of the green dyes after 10 min as shown in Figure 13. Additionally, Figure 14 showed low removal for the green dyes 2.1%. It means that the prepared nanoparticles of alumina are ineffective in sunlight. The comparison between the results of alumina nanoparticles and their nanocomposites indicated that the explosive technique is very effective for converting non-optical alumina to optical nanomaterials. By comparing with our previous results of zinc oxide nanoparticles, the prepared nanocomposites were more effective in sunlight than the most familiar optical materials because the Al doped ZnO nanoparticles caused reduction for the band gap energy from 3.29 to 3.23 eV leading to complete removal of NGB in 6 h under sunlight [21]. Recently, the complete decolorization and decomposition of green dye were achieved after 1.25–1.3 h of irradiation of UV light using the aluminium zinc oxide nanocomposite-coated CNTs and nanofibers [21,22].

## 3. Discussion

Conversion of aluminium oxides to photo-active materials was achieved through producing different kinds of carbon species during growth of aluminium oxides in the explosive reactions. In the case of using methanol as a source of carbon, CNTs were produced to build the first nanocomposite NC-1 and caused narrowing for the band gap energy to be 2.62 eV through creating new levels in the band gap region because of defect states of CNTs. The other two nanocomposites, NC-2 and NC-3, were prepared by using ethanol as a carbon source. Decomposition of ethanol led to growth of two types of carbon species, CNTs and thin film of carbon that combined with aluminium oxides nanoparticles to build two structures. Therefore, further narrowing occurred for the band gap energy of the nanocomposites NC-2 and NC-3 to become 1.6 eV because of the new structure of carbon; core-shell structure.

Depending on this observation, the decomposition of methanol is different from than that of ethanol. According to the heat of enthalpy of each alcohol, methanol is easily decomposed than ethanol because the standard heat of enthalpy of gas phase ethanol is −235.0 kJ/mol while the methanol has −201.0 kJ/mol [23]. Therefore, methanol is completely transformed to CNTs, while ethanol was converted to the main component, CNTs, and the secondary component, thin film of amorphous carbon.

The high performance of the prepared nanocomposites (NC-1, NC-2, and NC-3) as photocatalysts in sunlight could be explained by accelerating the excitation reaction because of the strong narrowing of the band gap energy of the prepared nanocomposites.

In the normal photocatalyst, the photocatalytic removal process depends on production of a group of reactions on its surface as shown in equations (4–6). When the photocatalyst is exposed to photons of energy equal to, or higher than, its band gap energy, electrons (e^•^) are excited and jumped to conduction band leaving holes (h^+^) in the valance band as shown in processes (4,7).

Excitation reaction:

Photocatalyst + Sunlight = e^•^ + h^+^ (hole) (4)

Accumulation process:

e^•^ = Conduction band (5)

Recombination reaction:

e^•^ + h^+^ = Photocatalyst (6)

In the case of using the prepared nanocomposites NC-1, NC-2, and NC-3, the excitation reaction is very fast because of their low band gap energy as shown in process (7). At the same time, the carbon nanotubes and core-shell carbon structure prevent both the accumulation process (5) and the recombination reaction (4) through the separation process (7) because the carbon is a good conductor for electrons. Accordingly, the production of strong oxidants is very fast as shown in equations (9,10). The strong oxidants completely destroy the green pollutants in a short time as seen in the processes (11,12).

Excitation reaction:

NC (1.6 eV) + Sunlight = e^•^ + h^+^ (hole) (7)

Separation process

e^•^ = Carbon species (8)

Production of Radicals:

OH^−^ + H^+^ = OH^•^ (9)

Production of super oxide radicals:

O_2_ + e^•^ = O_2_^•^ (10)

Degradation process:

NGB + O_2_^•^ = CO_2_ + H_2_O (11)

Degradation process:

NGB + OH^•^ = CO_2_ + H_2_O (12)

Finally, we can conclude that the prepared nanocomposites have a dual function for acting as photocatalysts. The first function is due to the strong narrowing of the band gap energy which is clearly observed for the nanocomposites NC-2 and NC-3. Therefore, these samples achieved 92–99% removal of pollutants because of accelerating the excitation reaction. The second function is owing to presence of two kinds of carbon species in the structure of these nanocomposites that prevented the electrons accumulation in the conduction band through quick transport of the excited electrons to the carbon surface to react with adsorbed dyes. For these reasons, these nanocomposites approximately caused a complete removal of green pollutants in ten minutes.

## 4. Materials and Methods

### 4.1. Preparation of Solid Fuel

A solid fuel consists of metallic fuel and explosive materials. Aluminium hydroxide can act as metallic fuel because it assists the explosive materials to become more energetic and aggressive combustion [24]. Aluminium nitrate is a well-known explosive [25]. It means that the combined structure between both aluminium hydroxide and ammonium nitrate can be considered a strong solid fuel. By using sol-gel method, aluminium hydroxide was prepared and saturated by ammonium nitrate. Typically, 500 milliliters of aqueous solution was prepared by dissolving 0.00015 mol of Cetyltrimethyl ammonium bromide and 0.03 mol of aluminum nitrate in deionized water. 10 wt.% of ammonium bicarbonate was used to precipitate white gel through slow addition to the aqueous solution of aluminium nitrate. By filtration, the white gel was collected and kept at 80 °C for drying under vacuum.

### 4.2. Preparation of Nanocomposites

The prepared dried gel was thermally treated at high pressure of alcohol to produce three nanocomposites (NC-1, NC-2, and NC-3) according to Table 1. By using methanol as a source for producing carbon species (carbon nanotubes and carbon nanorods), the first nanocomposite was prepared through explosive detonation process inside autoclave. 350 mL of methanol was mixed with 25 g of the dried gel which prepared in the first step. Then, the mixture was kept under pressure 10 bar and at temperature = 350 °C inside a closed vessel (autoclave). After 2 h, the system was shut down and the temperature started to decrease. In the same time, the autoclave pressure is decreased by gradually opening the valve of the autoclave. In order to remove the residual gases from the autoclave, it was fluxed with an inert gas (Argon). When the temperature of the autoclave attained to room temperature, the product was collected and called NC-1.

The second nanocomposite NC-2 was prepared by changing methanol to ethanol. By following the same procedure, 350 mL of ethanol was reacted with 25 g of the dried gel inside the autoclave through explosive detonation reaction. To indicate the effect of the ratio of ethanol and gel on the growth of carbon species, the third nanocomposite NC-3 was produced by reacting 350 mL of ethanol with 15 g of the dried gel inside the autoclave under the same conditions.

### 4.3. Characterization Techniques

To determine the crystalline structures of the prepared materials, powder X-ray diffraction technique was performed by Bruker-AXS, Karlsruhe, Germany with Cu-K_α_ radiation (λ = 0.154 nm). Electron Probe Micro analyzer JED 2300 (JEOL Company, Tokyo, Japan) was used to determine the different elements in the prepared materials through energy-dispersive X-ray spectroscopy technique. Thermogravimetric analyses were carried out by series Q500 of TA thermogravimetric analyzer (TA company, New Castle, PA, USA). Transmission Electron Microscopy with model JEM 2100F (JEOL Company, Tokyo, Japan) was used for imaging the prepared materials at the Nano scale. In order to identify the different kinds of the carbon species, Raman spectra were measured by Horiba-Jobin-Yvon instrument (Horiba Company, Montpellier, France) with a model Lab RAM HR Evolution equipped with a 633 ULF laser. The optical parameters of the prepared materials were measured by UV/VIS/NIR Shimadzu 3600 spectrophotometer (Shimadzu, Columbia, MD, USA) through the diffuse reflectance technique. To measure solid materials, an integrating sphere (ISR-603) was attached to the used spectrophotometer.

### 4.4. Photocatalytic Measurements

The photo activity of the prepared materials was tested through degradation of Naphthol Green B (NGB) in the sunlight. In the present study, 0.1 g of the prepared material is well-dispersed in the aqueous solution of NGB (4 × 10^−4^ M) and irradiated in sunlight. The irradiation area is 10 cm^2^. Depending on the law of Beer-Lambert [19], the concentration of the dye is proportional to the intensity of the measured spectrum of the dye when the initial concentration is low. By withdrawing a certain amount of the mixture after ten minutes of irradiation in sunlight, the absorbance of the diluted (typically 1:2 in water) samples were measured using UV-Vis spectrophotometer. The extent of decomposition of the green dyes is determined by calculating the integrated area of the characteristic peak of NGB at 714 nm. All photocatalytic degradation experiments were performed under irradiation of sunlight in the period of 9:30 A.M. and 10:30 A.M. during the summer season (July) in Saudi Arabia.

## 5. Conclusions

Many objectives have been achieved in the current study. The first objective was conversion of non-optical alumina to photoactive nanomaterials through explosive reactions. It was achieved by narrowing the band gap energy of aluminium oxide from 4.34 eV to 2.62 eV through growth of carbon nanotubes during explosive reactions. The second aim focused on converting alumina to be active in sunlight. It was accomplished by sharp reduction of the band gap energy to be 1.6 eV through modifying the structure of aluminium oxide by two different carbon species; CNTs and thin film of carbon. These carbon species were composed of core-shell structure and carbon nanotubes. The third objective was accelerating the degradation of industrial pollutants in the visible light. In this trend, 99% of green pollutants were destroyed after illuminating 10 min. in sunlight. Finally, based on the above results, we can conclude that the prepared nanocomposites are considered promising candidates for optical applications such as photocatalysis. In addition, these nanomaterials are suitable components for designing solar cells.

## Figures and Tables

**Table 1:** The used materials for preparing nanocomposites.

Sample	S. Carbon/S. Alumina Ratio *	Dried Gel	Alcohol
NC-1	11	25 g	Methanol
NC-2	11	25 g	Ethanol
NC-3	20	15 g	Ethanol

* S. carbon/S. alumina Ratio is the weight of source of carbon species to source of alumina.
